# The Oxidation Resistance of Nb-Si-Based Alloys at Intermediate and High Temperatures

**DOI:** 10.3390/ma13051229

**Published:** 2020-03-09

**Authors:** Jianyong Yang, Guanqun Zhuo, Kaiyong Jiang, Xinghan Zhu, Linfen Su

**Affiliations:** 1 College of Mechanical Engineering and Automation, Huaqiao University, Xiamen 361021, China; 2 Fujian Key Laboratory of Special Energy Manufacturing, Huaqiao University, Xiamen 361021, China

**Keywords:** oxidation, Nb-Si-based alloy, microstructure

## Abstract

The oxidation behavior of three Nb-Si-based alloys were evaluated at intermediate (800 °C) and high (1250 °C) temperatures for 100 h in air. At 800 °C, the Nb-24Ti-15Si-13Cr-2Al-2Hf (at. %) alloy suffered from catastrophic pest oxidation. This pest phenomenon was suppressed by the addition of Sn. However, Ta addition protected the Nb-Si-based alloys from pest oxidation for a short time. At 1250 °C, Sn could enhance the oxidation resistance of Nb-Si-based alloys due to the formation of a Sn-rich layer. In addition, the oxidation mechanisms of Nb-Si-based alloys at intermediate and high temperatures were discussed.

## 1. Introduction

Recently, the development of refractory intermetallic compounds (such as Nb_3_Al, Nb_5_Si_3_, Cr_2_Nb, MoSi_2_), which could operate at temperatures exceeding those tolerated by Ni-based superalloys, has been required in various fields [1,2,3]. Among them, Nb-Si-based alloys (consisting of Nb_5_Si_3_, niobium solid solution (Nb_SS_) and Cr_2_Nb) have been extensively studied due to their attractive high-temperature attributes, such as high melting points, acceptable room-temperature fracture toughness and excellent high-temperature strength [4,5,6]. 

However, the oxidation performance of Nb-Si-based alloys is a major drawback that has hindered their application [7,8,9]. Nb-Si-based alloys show serious levels of oxidation at both high (1000–1300 °C) and intermediate (600–900 °C) temperatures. At high temperatures, the rapid oxidation may be due to the formation of Nb_2_O_5_, which prevents the formation of a continuous silica layer [10,11]. At intermediate temperatures, Nb-Si-based alloys suffer from pest damage, resulting in the alloys disintegrating into powder [4,12,13,14]. 

Recently, alloying elements such as Ti, Cr, Al, Ge, B and rare elements have been added to enhance the oxidation behavior of Nb-Si-based alloys at high temperatures [15,16,17]. With increasing Si concentration, Nb-Si-based alloys show better oxidation performance [13,18]. Abundant Cr can promote the formation of the oxidation resistance phase Cr_2_Nb [19]. Furthermore, the oxidation resistance of Cr_2_Nb can be further enhanced by the addition of Al [20]. Additions of Ge and B promote the formation of a more compact and protective oxide scale, thus improving oxidation resistance [21]. A similar compact oxide scale is observed in Nb-Si-based alloys containing high levels of Al and Cr [22]. Small additions of rare earth elements can significantly enhance the oxidation resistance of metals as well as coatings [23,24,25]. An appropriate amount of Y (0.3 at. %) can refine alloys’ microstructure and enhance oxidation resistance [23]. However, the addition of Dy accelerates the oxidation rate of Nb-Si-based alloys and causes increased weight gain [24]. 

On the other hand, alloying is an effective way to eliminate the pest phenomenon of Nb-Si-based alloys at intermediate temperatures [5]. In the Mo-Si system, alloying with Al, Ti, Zr and Y can suppress the pest phenomenon due to the selective oxidation of alloying elements at the grain boundaries [26]. In the Nb-Ti-Si-Al-Cr system, Al reduces the pest susceptibility at 800 °C [27]. In the Nb-Ti-Si-Cr-Al-Mo system, 5 at. % of Sn can eliminate pest oxidation behavior at 800 °C [2]. Knittel et al. suggest that Nb-Si-based alloys show better oxidation resistance at 800 °C with increasing Sn concentration, but that a brittle phase (Nb,Ti)_3_(Sn,Ti) presents when the content of Sn is higher than 3 at. % [28].

In our previous study, a Nb-Si-based alloy consisting of Nb_SS_, Nb_5_Si_3_ and Cr_2_Nb showed acceptable high-temperature oxidation resistance [21], but its intermediate-temperature oxidation resistance was unclear. In this study, it was selected as the base alloy. Furthermore, to enhance both intermediate- and high-temperature oxidation resistance, 2 at. % of Sn and 2 at. % of Ta were added in the base alloy. The effects of Sn and Ta on the oxidation resistance of an Nb-Si-based alloys at intermediate (800 °C) and high (1250 °C) temperatures were investigated. In addition, the oxidation mechanisms of Nb-Si-based alloys at intermediate and high temperatures were discussed. 

## 2. Material and Methods

Three Nb-Si-based alloys with different compositions were prepared. The nominal compositions were Nb-24Ti-13Cr-2Al-2Hf-15Si (at. %), Nb-24Ti-13Cr-2Al-2Hf-15Si-2Sn (at. %) and Nb-24Ti-13Cr-2Al-2Hf-15Si-2Ta (at. %), respectively. The Nb-24Ti-13Cr-2Al-2Hf-15Si alloy was designed for comparison and was denoted as the base alloy. The Nb-24Ti-13Cr-2Al-2Hf-15Si-2Sn and Nb-24Ti-13Cr-2Al-2Hf-15Si-2Ta alloys were denoted as the 2Sn alloy and 2Ta alloy, respectively. They were fabricated by nonconsumable arc-melting. The ingots were remelted and inverted at least four times to ensure composition homogeneity. Oxidation samples with a size of 8 × 8 × 5 mm^3^ were cut from the centers of the ingots. All surfaces were mechanically grinded on wet SiC paper to 1200 grit.

The oxidation tests were conducted in an open-ended tube furnace in air. The oxidation temperatures were 800 and 1250 °C respectively. Each sample was placed in a separate alumina crucible. The samples were removed from the furnace at the intervals of 10, 20, 40, 60, 80 and 100 h and weighed together with the crucible using a precision analytical balance (Model CPA225D, Sartorius, Gottingen, Germany) with an accuracy of 0.00001 g. 

The phases of the three alloys and the oxide products were determined by X-ray diffraction (XRD, CuKa-radiation, X’Pert Pro, Panalytical, Almelo, Holland) in the range of 20–90° at a 2θ scanning rate of 6°/min. Cross-sections of the samples were grinded on wet SiC paper, starting with 800 grit and increasing to up to 4000 grit, and polished with diamond polishing paste (1 μm). Micrographs of cross-sections and surface morphologies of oxidized specimens were observed through a scanning electron microscope equipped with an energy-dispersive X-Ray spectroscopy system (Sigma 500, Zeiss, Oberkochen, Germany).

## 3. Results and Discussion

### 3.1. Microstructural Characterization of As-Cast Alloys

**Figure 1:**
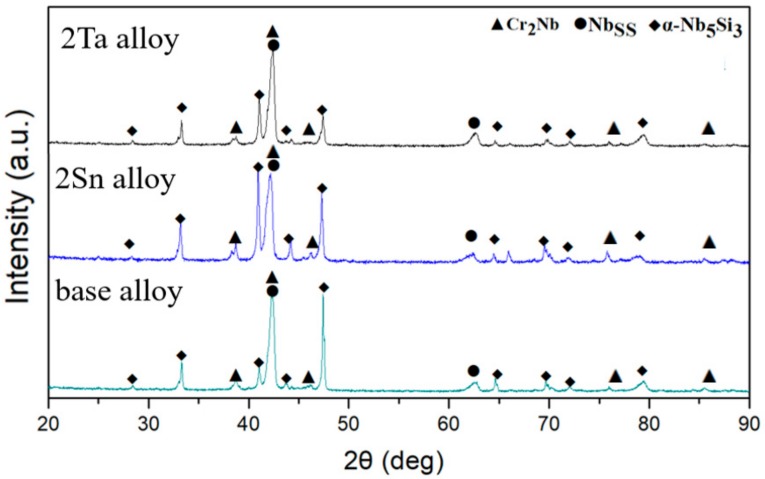
XRD patterns of the samples.

**Figure 2:**
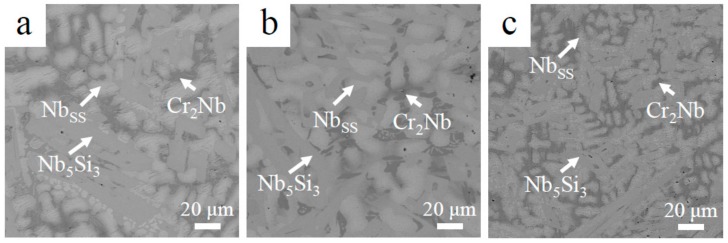
Microstructure of (**a**) the base alloy; (**b**) 2Sn alloy; (**c**) 2Ta alloy.

The XRD patterns of the base alloy, 2Sn alloy and 2Ta alloy are shown in Figure 1. Each of the three alloys consisted of Nb_SS_ (JCPDS card No. 35-0789), Nb_5_Si_3_ (JCPDS card No. 30-0875) and Cr_2_Nb (JCPDS card No. 47-1638) phases. The XRD results indicated that the constituent phases did not change, as the base alloy alloyed with 2 at. % of Ta or 2 at. % of Sn. Figure 2 demonstrates the microstructures of the three Nb-Si-based alloys. The microstructure of the base alloy consisted of primary Nb_5_Si_3_, eutectic (Nb_SS_ + Nb_5_Si_3_) and Cr_2_Nb. The addition of 2 at. % of Sn enlarged the size of the Nb_SS_ dendrites. The addition of 2 at. % of Ta refined the size of both the Nbss dendrites and the Nb_5_Si_3_ blocks.

### 3.2. Intermediate Temperature Oxidation Resistance

**Figure 3:**
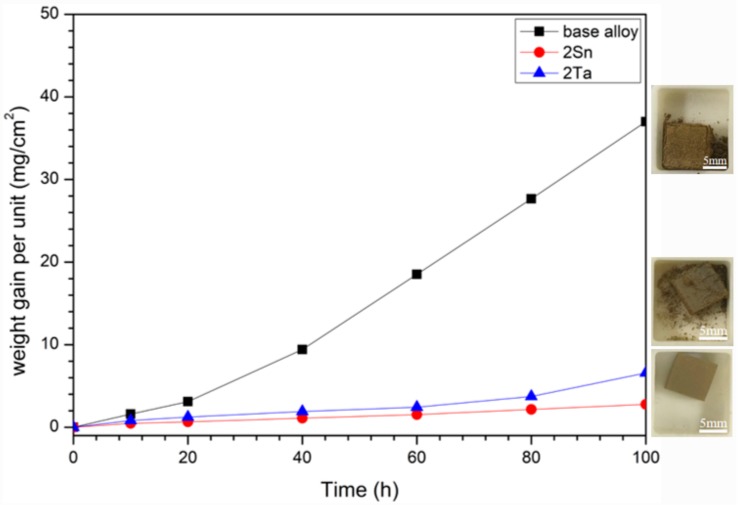
Weight gain versus time for base, 2Sn and 2Ta alloys oxidized at 800 °C for 100 h.

Figure 3 demonstrates the oxidative weight-gain curves and photographs of the oxidized alloys at 800 °C for 100 h. As shown in Figure 3, the base alloy showed a linear oxidation behavior over the first 20 h, then accelerated oxidation behavior was observed after 40 h; after 100 h at 800 °C, the weight gain was 36.6 mg/cm^2^. In the 2Ta alloy, the accelerated oxidation behavior was observed after 80 h; after 100 h at 800 °C, the weight gain was 6.58 mg/cm^2^. Noteworthily, the weight gain of the 2Sn alloy was 2.78 mg/cm^2^, which was only one sixth of that of the base alloy. As shown in Figure 1, the base alloy and the 2Ta alloy both degraded into powder, suggesting that catastrophic pest oxidations had occurred. The oxide scale of the 2Sn alloy was tightly adherent, indicating that the sample was protected upon oxidation. These results suggest that Sn plays a crucial role in suppressing the pest oxidation phenomenon of Nb-Si-based alloys at intermediate temperatures.

In general, oxidation kinetics of 2Sn and 2Ta alloys are calculated by the following formula [17,29]:

(1) $$\frac{\Delta m}{S}={\left(k\cdot t\right)}^{n}$$

where Δ*m, S, t* and *n* are the mass variation, the total surface area of the sample, the oxidation rate coefficient and the oxidation duration rate exponent, respectively. The oxidation duration rate exponent *(n*) of the 2Sn alloy and the 2Ta alloy at 800 °C were determined to be 0.84 and 0.83 respectively, by fitting the thermal gravimetric data according to Formula (1). Therefore, the oxidation kinetics of both the 2Sn and the 2Ta alloys at 800 °C followed a parabolic-linear law. The oxidation duration-rate exponents of 2Sn and 2Ta alloys were close to 1, suggesting that the surface reactions were the dominant rate-determining step for the oxidation; that is, the rate of the interfacial reaction of O_2_ with Nb-Si-based alloys. This oxidation behavior suggests that the oxidation products had a slight effect on the oxidation rate. 

**Figure 4:**
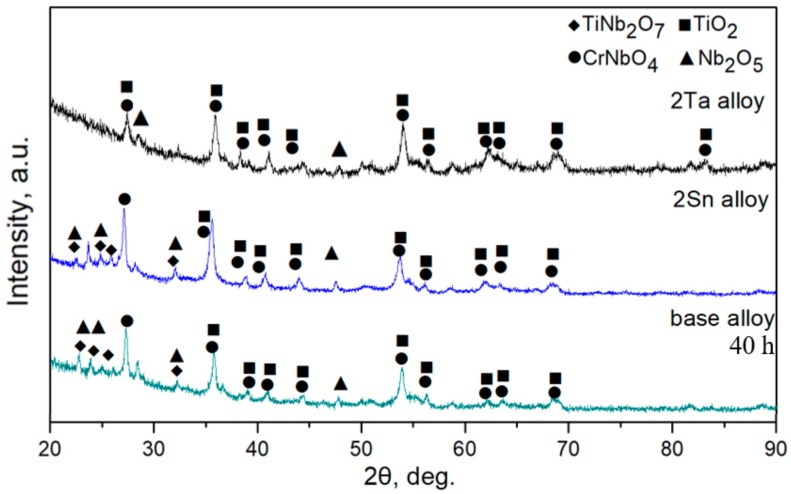
XRD patterns of oxides obtained after oxidation at 800 °C for 100 h.

**Figure 5:**
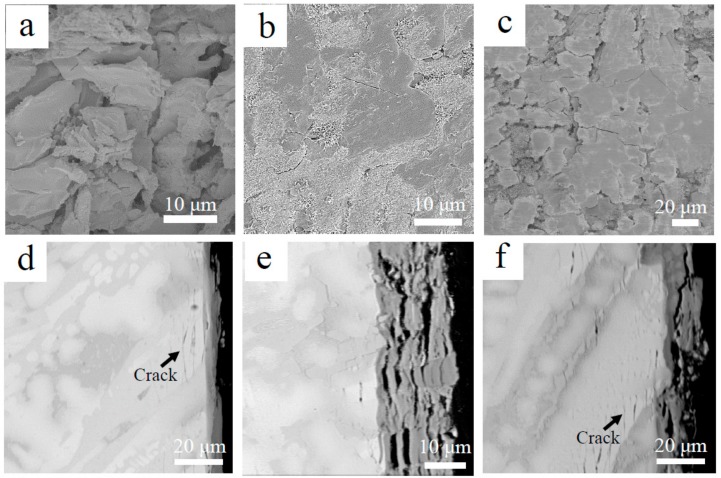
Surface (**a**–**c**) and cross-sectional (**d**–**f**) morphologies of Nb-Si-based alloy after oxidation at 800 °C for 100 h: (**a**,**d**) base alloy (40 h); (**b**,**e**) 2Sn alloy; (**c**,**f**) 2Ta alloy.

Figure 4 demonstrates XRD patterns of oxidized products of the three Nb-Si-based alloys after oxidation at 800 °C. The results showed that oxidized products mainly consisted of Nb_2_O_5_ (JCPDS card No. 32-0711)_,_ TiO_2_ (JCPDS card No. 21-1276), TiNb_2_O_7_ (JCPDS card No. 39-1407) and CrNbO_4_ (JCPDS card No. 34-0366). Surface and cross-sectional morphologies of the three alloys after oxidation at 800 °C are shown in Figure 5. Unlike the rough and porous surface morphology of the base alloy (Figure 5a), the appearance of 2Sn alloy (Figure 5b) showed that its surface remained basically intact. The surface of the 2Ta alloy (Figure 5c) remained largely intact, but a lot of cracks were found on the surface. The oxide scale of the base alloy (as shown in Figure 5d) was thin, due to serious spallation. Although the oxide scale of the 2Sn alloy (as shown in Figure 5e) was intact, the oxide scale of the 2Ta alloy (as shown in Figure 5f) was cracked, which may have been due to metallographic preparation. Moreover, some cracks formed on the Nb_5_Si_3_ near the interface of oxide and substrate in the base and 2Ta alloys. However, only a few cracks formed on the Nb_5_Si_3_ in the 2Sn alloy. The inward diffusion of oxygen led to the volume expansion of Nb_SS_, inducing tensile strains to silicides at intermediate temperatures [30]. Therefore, cracks were formed on Nb_5_Si_3_ after oxidation. 

**Figure 6:**
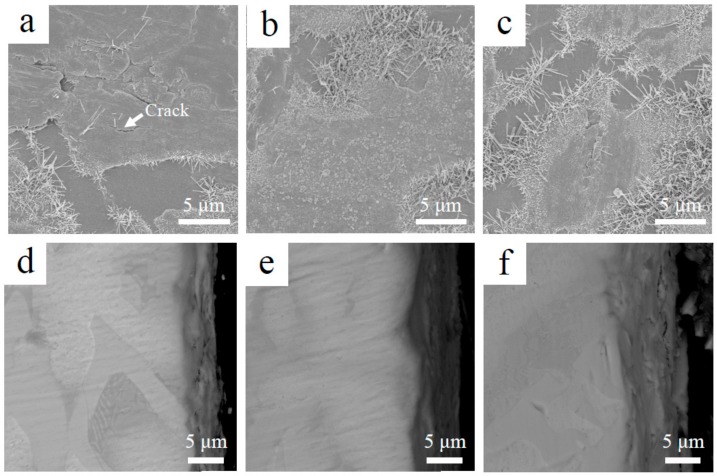
Surface (**a**–**c**) and cross-sectional (**d**–**f**) morphologies of Nb-Si-based alloys after oxidation at 800 °C for 10 h: (**a**,**d**) base alloy; (**b**,**e**) 2Sn alloy; (**c**,**f**) 2Ta alloy.

**Figure 7:**
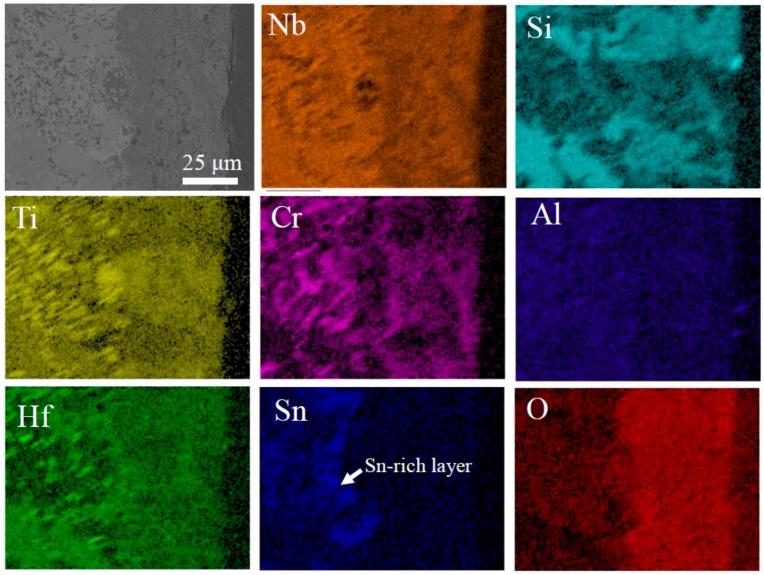
Oxide metal interface in Nb-24Ti-13Cr-2Al-2Hf-15Si-2Sn alloy oxidized at 800 °C for 10 h.

To reveal the short-term oxidation behavior of Nb-Si-based alloys at 800 °C, oxidation tests were conducted at 800 °C for 10 h. Figure 6 shows the surface and cross-sectional microstructure of the three alloys after oxidation at 800 °C for 10 h. The images indicate that the oxide scales of all three alloys remained basically intact. The rod-like oxide and glassy oxide were formed from Nb_SS_ and Nb_5_Si_3_ respectively. The thickness of oxide scales of the base alloy, 2Sn alloy and 2Ta alloy are 5, 6 and 8 μm respectively. To indicate the elemental analysis in the scale, the X-ray mapping of the 2Sn alloy after oxidation at 800 °C for 10 h is shown in Figure 7. The results clearly showed the presence of O, Nb, Ti, Si, Al, Hf, Sn and Cr in the oxides. Nb, Al, Ti, Cr and Hf were almost uniformly distributed at the scale. A Sn-rich layer was observed between the oxide scale and substrate. Furthermore, Si was enriched in the outer layer of the oxide scale, suggesting that a SiO_2_ layer had formed. A similar SiO_2_ layer was also observed in the 2Ta alloy.

Some cracks were observed in the surface of the base alloy (as shown in Figure 6a) after oxidation at 800 °C for 10 h. This would have provided more sites for rapid inward diffusion of oxygen, resulting in the higher oxidation rate. Therefore, the pest oxidation of the base alloy may have been due to the generation of cracks in the brittle Nb_5_Si_3_. The formation of cracks increases the oxygen intake rate and leads to catastrophic oxidation behavior. As suggested by Mathieu et al., the crack formation mechanism of Nb-Si-based alloys at medium temperatures is due to the progressive volume expansion of oxides in Nb_SS_ during oxidation [30]. Due to this mechanism, pesting can be eliminated by limiting the inward diffusion of oxygen in Nb_SS_. In the 2Sn and 2Ta alloys, a SiO_2_ layer developed after oxidation (Figure 7). This silica layer reduced the inward oxygen diffusion rate, contributing to the enhancement of the oxidation resistance [23,31]. We therefore deduced that the interdiffusion rate of oxygen in the 2Sn and 2Ta alloys were lower than that of the base alloy. The formation of cracks was consequently suppressed, leading to enhanced oxidation resistance. In addition, the oxidation rate of the 2Ta alloy was higher than that of the 2Sn alloy. The 2Ta alloy suffered from accelerated oxidation beyond 80 h, suggesting that the addition of Ta protects Nb-Si-based alloys from oxidation for a short time.

In addition, the Sn-rich layer in the 2Sn alloy acted as a diffusion barrier against oxygen. Sn accumulated at the region between oxide scale and substrate due to its very low affinity for oxygen, compared to the other constitutive elements of Nb-Si-based alloys [28]. Due to the SiO_2_ and Sn-rich layers, the interdiffusion rate of oxygen in the 2Sn alloy was lowest. Therefore, the pest phenomenon was suppressed by the addition of Sn.

### 3.3. High Temperature Oxidation Resistance

**Figure 8:**
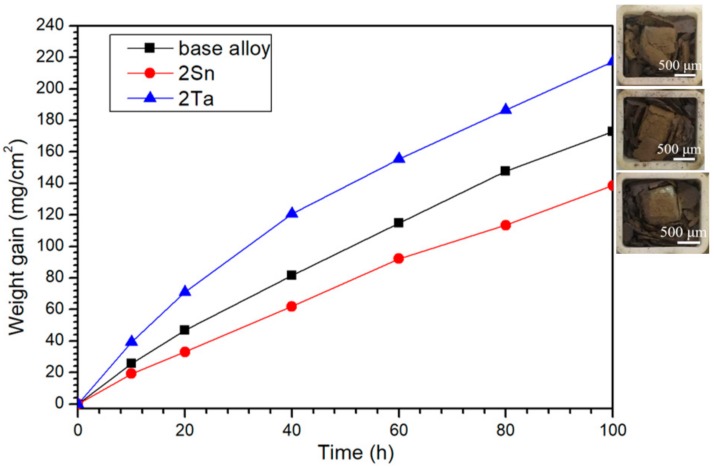
Weight gain versus time for base: 2Sn and 2Ta alloys oxidized at 1250 °C for 100 h.

Figure 8 shows the oxidative weight-gain curves and photographs of the oxidized alloys at 1250 °C. The weight gains of the base alloy, 2Sn alloy and 2Ta alloy after oxidation at 1250 °C for 100 h were 173.0, 138.6 and 217.2 mg/cm^2^, respectively. All three of the samples suffered severe spallation of oxide scales. Noteworthily, 2 at. % of Sn decreased the weight gain by approximately 19.9%, indicating the positive effects of Sn on the oxidation behavior of Nb-Si-based alloys at high temperatures. However, the addition of 2 at. % of Ta increased the weight gain by approximately 25.6%, suggesting that Ta has a detrimental effect on high temperature oxidation performance. 

According to Formula (1), oxidation duration rate exponents of the base alloy, 2Sn alloy and 2Ta alloy at 1250 °C were calculated to be 0.82, 0.69 and 0.86, respectively. Thus, the oxidation behavior of all three of the alloys followed a mixed parabolic-linear law at 1250 °C. This behavior suggested that the oxidation was governed by both interface reaction and diffusion. Furthermore, the oxidation duration rate exponents of the base, 2Sn and 2Ta alloys were close to 1, thus surface reaction was the dominant rate-determining step. 

**Figure 9:**
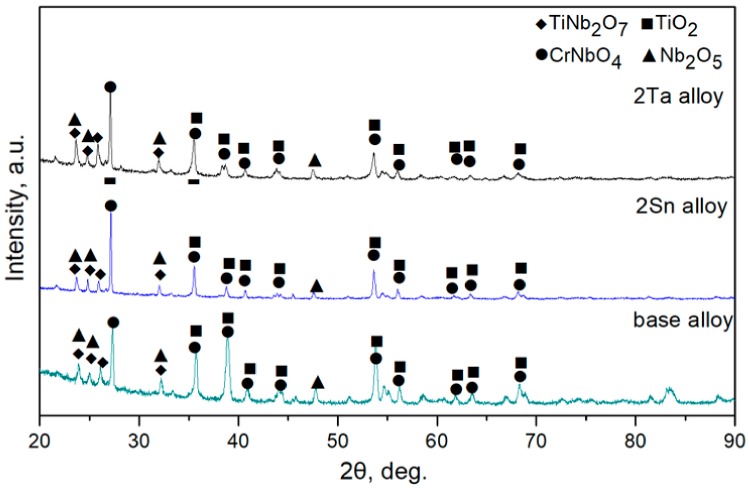
XRD patterns of oxides obtained after oxidation at 1250 °C for 100 h.

**Figure 10:**
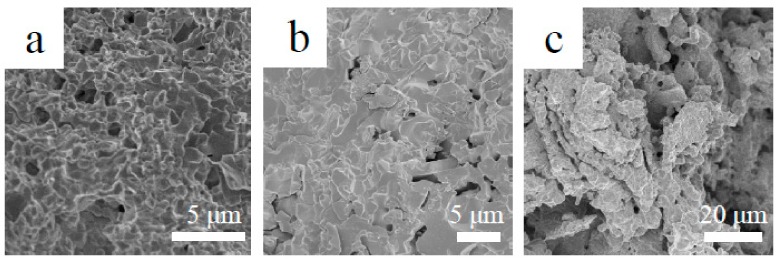
Surface morphologies of Nb-Si-based alloys after oxidation at 1250 °C for 100 h: (**a**) base alloy; (**b**) 2Sn alloy; (**c**) 2Ta alloy.

Figure 9 demonstrates XRD patterns of oxidized products formed on the three Nb-Si-based alloys after oxidation at 1250 °C. The oxidized products were TiNb_2_O_7_, CrNbO_4_, Nb_2_O_5_ and TiO_2_ phases. The oxide products were in good agreement with the oxidation of a Nb-24Ti-2Hf-6Cr-6Al-16Si (at. %) at 1250 °C obtained by TEM and selective-area diffraction. Figure 10 shows the residual oxide scale morphologies after oxidation at 1250 °C for 100 h. The oxide scales formed on the three Nb-Si-based alloys were rough and porous. 

**Figure 11:**
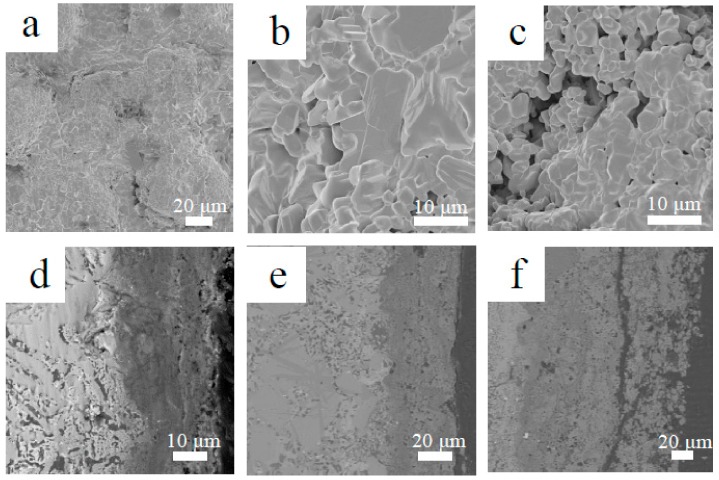
Surface (**a**–**c**) and cross-sectional (**d**–**f**) morphologies of Nb-Si-based alloys after oxidation at 1250 °C for 10 h: (**a**,**d**) base alloy; (**b**,**e**) 2Sn alloy; (**c**,**f**) 2Ta alloy.

To reveal the short-term oxidation behavior of Nb-Si-based alloys at 1250 °C, oxidation tests were conducted at 1250 °C for 10 h. Figure 11 demonstrates the surface morphologies and cross-sectional microstructures of the three alloys after oxidation at 1250 °C for 10 h. These images indicate that the oxide scales of the three alloys remained basically intact. The thickness of oxide scales of the base alloy, 2Sn alloy and 2Ta alloy were about 20, 40 and 120 μm respectively. Cracks formed in the 2Ta alloy, which may have been due to the growing stress of oxide scale. This cracking may have led to the spalling of oxide scales. As shown in Figure 11, the thickness of the 2Ta alloy was three times that of the 2Sn alloy after oxidation for 10 h (Figure 11e,f), which suggests that the addition of Ta led to faster inward transportation of oxygen. Therefore, Ta addition has detrimental effects on the oxidation resistance of Nb-Si-based alloys. 

**Figure 12:**
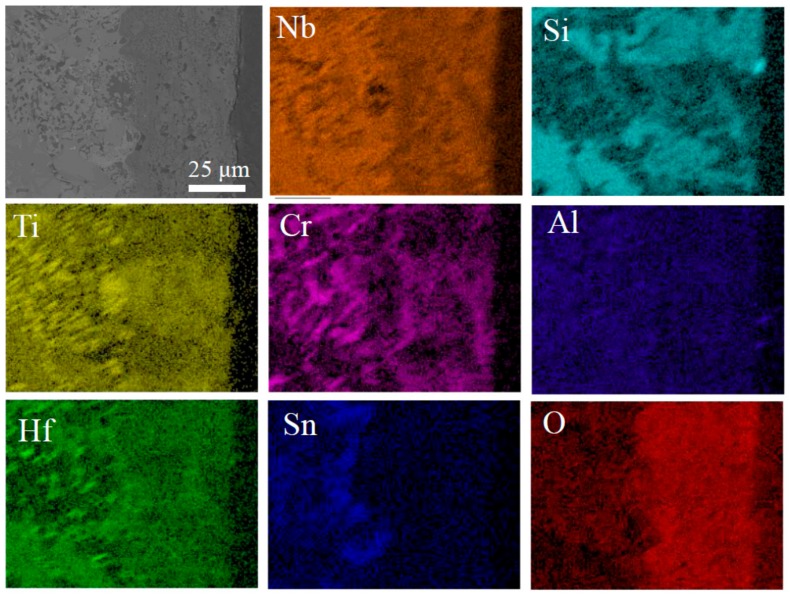
Oxide metal interface in Nb-24Ti-13Cr-2Al-2Hf-15Si-2Sn alloy oxidized at 1250◦C for 10 h.

An X-ray mapping of the 2Sn alloy after oxidation at 1250 °C for 10 h is shown in Figure 12. The results clearly demonstrated the presence of O, Nb, Ti, Si, Al, Hf, Sn and Cr in the oxides. Nb, Al, Ti, Cr and Hf were almost uniformly distributed at the scale. A Sn-rich layer formed between the oxide scale and substrate. This layer acted as a diffusion barrier against oxygen, leading to a lower inward diffusion rate of oxygen [28]. Therefore, the 2Sn alloy showed the best oxidation resistance in this study.

Furthermore, the three alloys were fully affected by internal oxidation just after oxidation at 1250 °C for 10 h. The internal oxides TiO_2_ with black contrast and HfO_2_ with white contrast mainly distributed in Nb_SS_ and the interface between Nb_SS_ and Nb_5_Si_3_(Cr_2_Nb). At high temperatures, the inward diffusion of oxygen mainly occurred through Nb_SS_, due to the faster transport of oxygen in Nb_SS_ than that in Cr_2_Nb and Nb_5_Si_3_ [4,10].

As revealed by the XRD results (Figure 9), the oxide scales that formed on the base alloy, 2Sn alloy and 2Ta alloy at 1250 °C mainly consisted of TiO_2_, Nb_2_O_5_ and TiNb_2_O_7_ and CrNbO_4_. The formation of Nb_2_O_5_ induced extensive compressive stress in the oxide scale, leading to the formation of cracks in oxide scales [20]. Moreover, the glassy SiO_2_ resulting from the decomposition of silicides was limited due to its insufficient volume. Thus, the glassy SiO_2_ phase could not heal all the cracks. Resultantly, the unprotective oxide scale led to the continuous diffusion of oxygen through the Nbss phases, thus the substrate suffered from a mixed parabolic-linear degradation.

In addition, the difference in the thermal expansion of the Nb-Si-based alloys and oxides generated thermal stress during cooling. The thermal stress also induced the cracking of oxide scale. Moreover, exposure to the cyclic temperatures generated more stresses than isothermal exposure. Unfortunately, according to Figure 11, the addition of Ta or Sn did not significantly reduce stress generation in the oxide scales.

## 4. Conclusions

The effects of Sn and Ta on the oxidation behavior of Nb-Si-based alloys were investigated at 800 °C and 1250 °C.

1. The microstructures of the base alloy, 2Sn alloy and 2Ta alloy consisted of Nb_5_Si_3_, Nb_SS_ and Cr_2_Nb.
2. The base alloy suffered from catastrophic pest oxidation at 800 °C. The addition of Sn suppressed the pest phenomenon. However, the addition of Ta protected the Nb-Si-based alloy from pest oxidation for a short time.
3. At 1250 °C, 2 at. % of Sn could enhance the oxidation resistance of Nb-Si-based alloys due to the formation of a Sn-rich layer. However, 2 at. % of Ta facilitated the a faster transportation of oxygen, resulting in worse oxidation resistance.
